# Expression of calpastatin hcast 3-25 and activity of the calpain/calpastatin system in human glioblastoma stem cells: possible involvement of hcast 3-25 in cell differentiation

**DOI:** 10.3389/fmolb.2024.1359956

**Published:** 2024-07-30

**Authors:** Sonia Spinelli, Federica Barbieri, Monica Averna, Tullio Florio, Marco Pedrazzi, Beatrice F. Tremonti, Michela Capraro, Roberta De Tullio

**Affiliations:** ^1^ IRCCS Istituto Giannina Gaslini, Laboratory of Molecular Nephrology, Genova, Italy; ^2^ Department of Experimental Medicine (DIMES), Section of Biochemistry, University of Genova, Genova, Italy; ^3^ Department of Internal Medicine (DIMI), Section of Pharmacology, University of Genova, Genova, Italy; ^4^ IRCCS Ospedale Policlinico San Martino, Genova, Italy

**Keywords:** hcast 3-25, calpastatin, calpain, glioblastoma stem cell, differentiation

## Abstract

Glioblastoma (GBM) is the most malignant brain tumor, characterized by cell heterogeneity comprising stem cells (GSCs) responsible for aggressiveness. The calpain/calpastatin (calp/cast) proteolytic system is involved in critical physiological processes and cancer progression. In this work we showed the expression profile of hcast 3-25 (a Type III calpastatin variant devoid of inhibitory units) and the members of the system in several patient-derived GSCs exploring the relationship between hcast 3-25 and activation/activity of calpains. Each GSC shows a peculiar calp/cast mRNA and protein expression pattern, and hcast 3-25 is the least expressed. Differentiation promotes upregulation of all the calp/cast system components except hcast 3-25 mRNA, which increased or decreased depending on individual GSC culture. Transfection of hcast 3-25-V5 into two selected GSCs indicated that hcast 3-25 effectively associates with calpains, supporting the digestion of selected calpain targets. Hcast 3-25 possibly affects the stem state promoting a differentiated, less aggressive phenotype.

## Introduction

**FIGURE 1:**
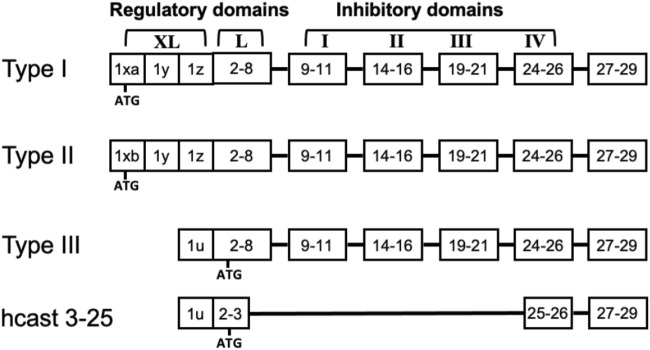
Structure of full length calpastatins and hcast 3-25. The schematic representation of the domains in the different calpastatin isoforms includes the position of the first ATG. Numbers indicate the exons present in each domain. Although hcast 3-25 sequence includes exons 25-26 of the fourth inhibitory domain, the consensus sequence «TIPPEY» for the inhibition of calpain is immediately upstream of the splicing site connecting exon 3 to exon 25.

The calpain-calpastatin (calp/cast) system is constituted by a family of intracellular Ca^2+^-dependent cysteine proteases and a single natural inhibitor called calpastatin (Goll et al., 2003). Among the multiple calpain forms present in mammals, six are tissue-specific, and the others are expressed across all tissues, including CNS (Ono and Sorimachi, 2012). The most studied are the ubiquitous calpain-1 (Calp-1) and calpain-2 (Calp-2), which were previously indicated as μ- and m-calpain based on their [Ca^2+^] requirement for activation *in vitro*. Both Calp-1 and Calp-2 are heterodimers containing a common 28-kDa regulatory subunit (calpain small subunit 1, CSS1, also named CAPN4) and a large 80-kDa catalytic subunit (CAPN1 and CAPN2, respectively) which shares 55%–65% sequence homology between the two enzymes. An increase in [Ca^2+^]_i_ promotes the dissociation of the heterodimer and activation by autoproteolysis of the protease. This same autolysis reduces the mass of both Calp-1 and Calp-2 catalytic subunits to 76-kDa and 78-kDa, respectively, and CSS1 mass to 18-kDa (Cong et al., 1989; Ono and Sorimachi, 2012). Calpastatin (Cast) is the specific inhibitor of the ubiquitous calpains and, although it exists as a single gene [in humans ref. Inazawa et al. (1990)], multiple protein forms resulting from both the use of different promoters (Lee et al., 1992; Takano et al., 2000; Raynaud et al., 2005) and alternative splicing mechanisms are produced (Lin et al., 2004; De Tullio et al., 2007). Full-length Cast polypeptide consists of four repetitive inhibitory domains and an NH_2_-region containing XL- and/or L-domain, which has no inhibitory activity and undergoes alternative splicing (Figure 1). Cast can also be selectively cleaved by calpain in fragments retaining inhibitory activity. Such a process leads to the amplification of the inhibitory effect of Cast, a condition required to block and regulate the activated calpain molecules generated following [Ca^2+^]_i_ increase (De Tullio et al., 2000; Stifanese et al., 2010). In normal conditions the efficient control of calpain activation/activity is also achieved through cytosolic diffusion of calpastatin in colocalization with calpain (De Tullio et al., 2009) and an increase in the inhibitor biosynthesis (Stifanese et al., 2010).

Ubiquitous calpains in addition to being involved in normal processes of neurogenesis, cell proliferation, differentiation, long-term potentiation, and memory (Amini et al., 2013; Machado et al., 2015; Wang et al., 2020; Baudry et al., 2021), play a role in various CNS pathologies generally associated to altered Ca^2+^-homeostasis (Stifanese et al., 2010; Rao et al., 2016; Mahaman et al., 2019; Muruzheva et al., 2021). In this context, the role of calpains has been studied through inhibition of calpain activity either by overexpressing calpastatin (Schoch et al., 2012; Yu et al., 2020) or using synthetic calpain inhibitors (Kerstein et al., 2017; Hu et al., 2019; Ivleva et al., 2022). Calp-1 or Calp-2 knockout models were used to explore the role of a specific calpain isoform in the CNS (Wang et al., 2020; Baudry et al., 2021; Wang et al., 2021). Calpains are also involved in the development and progression of different cancer types including glioblastoma (GBM) (Storr et al., 2011a; Moretti et al., 2014; Nian and Ma, 2021; Shapovalov et al., 2022), the most common and malignant adult brain tumor, a deadly disease without effective treatment (Ostrom et al., 2019; Tan et al., 2020). GBM is characterized by high cellular heterogeneity consisting of various cell populations including glioblastoma stem cells (GSCs), intrinsically resistant to chemo- and radio-therapy, sustaining cancer initiation, maintenance and recurrence (Bao et al., 2006). GSCs are able to self-renew and differentiate into a heterogeneous progeny of differentiated non-stem GBM cells, constituting the tumor bulk. GSCs and more differentiated GBM cells are two phenotypic states functionally defined, with distinct transcriptional, epigenetic, and metabolic profiles leading to different proliferation rates, migratory ability and chemo-sensitivity (Gimple et al., 2019). However, since plasticity of GBM cell populations is bidirectional and mainly modulated by environmental factors, successful GSC eradication should be paralleled by non-GSCs impairment. Deciphering novel potential molecular pathways, such as those involving the calp/cast system sustaining GBM cell stemness, could prospectively serve as prognostic and/or therapeutic factors for GBM. For example, recent findings suggest that calpain-2 contributes to temozolomide (the first-line chemotherapy drug for GBM patients) resistance in GBM cells, opening future implications for GBM patient management (Stillger et al., 2023).

Upregulation of Calp and Cast mRNAs was first reported in a single GBM compared to normal brain tissue (Ray et al., 2002). In a recent paper, Guo et al. (2022) demonstrated that in human glioblastoma cells, Calp-1, through the degradation of T291-phosphorylated IkBα, leads to NF-kB activation, which, in turn, by upregulating PD-L1 expression, results in increased tumor immune evasion (Guo et al., 2022). In this context, the phosphorylation of IkBα is catalyzed by hexokinase-2 (HK2), a glycolytic enzyme that was found overexpressed in numerous types of cancer (Garcia et al., 2019). Overexpression of CSS1, described in glioma tissues, was associated with low miR-124 expression and proposed as a potential adverse prognostic marker (Cai et al., 2014). CSS1 downregulation or miR-124 mimics suppressed migration and invasion of GBM cell lines (Cai et al., 2016). In zebrafish, Calp-2 expression is involved in GBM cell brain invasion (Lal et al., 2012). Vo et al. (2019a) demonstrated that a member of the nuclear factor I family, NFI, involved in GBM cell migration and infiltration, regulates the expression of the various Cast transcripts (i.e., Type III) by targeting the usage of alternative promoters in the CAST gene. The phosphorylation of NFI is regulated by the phosphatase calcineurin which is activated by calpain-mediated digestion (Vo et al., 2019b). Calpain exerts antiproliferative and antimigratory effects in established GBM cell lines by inducing the cleavage of filamin A (Cai et al., 2020), while Cast phosphorylation promotes radiation resistance in GSCs by increasing the activity of calpains (Bassett et al., 2018).

We have previously identified a new Cast variant belonging to Type III isoform (GenBank MK585065) but lacking the inhibitory units in a glioblastoma, a meningioma and four breast cancer biopsies as well as in PBMC from healthy donors (Sparatore et al., 2019). This “anomalous” Cast was named hcast 3-25 because the sequence of its transcript includes exon 2 and 3 of the L-domain and then “skips” into exon 25 immediately after the fourth inhibitory domain without changing the reading frame (Figure 1). Our *in vitro* experiments using the recombinant protein evidenced that hcast 3-25 associates with native Calp-1 in the absence of Ca^2+^ and protects the activated form from degradation when Ca^2+^ is present (Sparatore et al., 2019).

All these observations prompted us to explore if hcast 3-25 could be included as a new member in the calpain/calpastatin system acting as a positive modulator of calpains in GBM exploiting the availability of several fully characterized patient-derived GBM stem cultures.

In the present study we investigated a possible correlation between the expression of hcast 3-25 and the other components of the calp/cast system in a set of matched stem and differentiated GBM cultures. We determined the mRNA and protein levels of Calp-1, Calp-2, CSS1 and Cast and the expression of hcast 3-25 has been evaluated in comparison with the other full-length Casts using primers spanning the specific XL-L region of each Cast isoform. We assessed the activation of the calp/cast system in cell crude lysates by detecting both Calp-1 and Calp-2 autoproteolyzed forms and Cast as well as CSS1 species resulting from calpain-mediated cleavage. The activity of calpains has been evaluated as the production of the specific calpain-derived α-II spectrin fragments (Czogalla and Sikorski, 2005). On this basis, new insights into the calp/cast system in GBM stemness or the phenotypic shift to a more differentiated state may identify novel molecular vulnerabilities of GBM cells.

## Materials and methods

### Human astrocytoma cell lines

Three established human cell lines were used: the high grade malignant glioma U87-MG (GBM WHO IV), and low-grade human pediatric astrocytoma Res186 (pilocytic astrocytoma grade I) and Res259 (astrocytoma grade II). All cultures were maintained in DMEM supplemented with 10% fetal bovine serum (FBS), 2 mM L-glutamine, 100 μg/mL penicillin-streptomycin (all from EuroClone) under atmosphere controlled at 5% CO_2_.

### GBM primary cultures, GBM stem cells and differentiation

GBM primary cell cultures were immediately isolated from explanted tumors and characterized as previously detailed (Griffero et al., 2009; Wurth et al., 2013). All GBMs were newly diagnosed: clinico-histopathological features of patients and tumors are reported in Supplementary Table S1. Cells (GBM stem cells, GSCs) were grown in a serum-free stem-permissive medium: 1:1 DMEM-F12/Neurobasal™ (Gibco-ThermoFisher Scientific), B27™ supplement (Gibco-ThermoFisher Scientific), 2 mM L-glutamine (EuroClone), 1% penicillin-streptomycin (EuroClone), 15 μg/mL insulin (Sigma-Aldrich), 2 μg/mL heparin (Sigma-Aldrich), recombinant human bFGF (10 ng/mL; Miltenyi Biotec) and EGF (20 ng/mL; Miltenyi Biotec). Under these conditions cells grow as floating spheres. Stem cultures were inspected daily to check sphere formation and density. They were split 1:3 to 1:5 depending on cell density and speed of growth. To obtain single cell suspension, neurospheres necessary for experiments were disaggregated with TrypLE Express (Thermofisher Scientific) and gentle pipetting. To perform experiments, and improve feasibility and reliability, cells were grown on growth factor-reduced Matrigel™-coating (1:200, Corning, ThermoFisher Scientific) as monolayers, maintaining intact self-renewal ability, stem cell marker expression, and tumorigenicity by orthotopic xenograft in NOD/SCID mice, injecting 10,000 cells (see Supplementary Table S1) (Griffero et al., 2009). Monolayer growth in stem medium neither induces differentiation program nor affects spherogenic properties once cells are cultured without Matrigel coating. Non-stem differentiated GBM cells were obtained removing, bFGF, EGF and B27 supplement from medium, and growing cells in DMEM-F12 medium supplemented with 10% FBS (Gibco), 2 mM  L-glutamine, 1% penicillin-streptomycin for 2  weeks (Gatti et al., 2013; Barbieri et al., 2022). Morphological changes were monitored and imaged by inverted microscope (Leica).

### RNA isolation, cDNA synthesis and RT-qPCR analysis

Total RNA was isolated from human GSCs and GBM cell lines (1 × 10^6^ cells) using RNeasy Kit (Qiagen) according to the manufacturer’s instructions. cDNA was obtained from 5 μg of total RNA with oligo (dT)20 primer using the Superscript IV RT-PCR system (Invitrogen, Thermo Fisher Scientific). To obtain the highest yields and very long RNA transcripts, cDNA synthesis was carried out at 50°C for 10 min followed by 30 min incubation at 65°C. A final step at 80°C for 10 min was performed to stop the reaction.

RT-qPCR analysis was carried out using iQ5 Real-Time PCR detection system (Bio-Rad), the human-specific primers were designed using Beacon Designer 2.0 software (Bio-Rad) and sequences are listed in Supplementary Table S2. Each sample was assayed in triplicate in a 10 μL amplification reaction, containing 14 ng of cDNA, the specific primer mixture (0.4 μM each of sense and antisense primers) and 5 μL of 2X iQ SYBR Green Supermix Sample (Bio-Rad) (Spinelli et al., 2021). The amplification program included 40 cycles and the PCR conditions were: 95°C 30 s, 60 or 62°C (depending on the gene, see Supplementary Table S2) 20 s, and 72°C 30 s. Fluorescence products were detected at the last step of each cycle. To verify the purity of the products, a melting curve was produced after each run. Stably expressed ribosomal protein lateral stalk subunit P0 (RPLP0) was used as housekeeping gene. Statistical analysis was performed using the iQ5 Optical System Software version 1.0 (Bio-Rad Laboratories) based on the 2^-△△^Ct method. The dissociation curve for each amplification was analyzed to confirm the absence of nonspecific PCR products.

### RNA sequencing

GSC transcriptome was studied by RNA sequencing (RNA-seq) as previously reported (Barbieri et al., 2022). Briefly, RNA obtained from GSC cultures was checked for quality with Agilent TapeStation, and library preparation was carried out with Truseq stranded mRNA (Illumina), sequenced on Illumina platform. Up to 80M reads were obtained for each sample. Reads were aligned with STAR 2.7.5c and counted with RSEM 1.3.2 (Dobin and Gingeras, 2015). Data have been deposited in GEO repository: GEO accession GSE179356.

### Preparation of crude protein extracts from cell cultures and western blot

Cell pellets (2 × 10^6^), stored at −80°C, were suspended in ice-cold (200 μL) 50 mM Na Borate buffer, pH 7.5, containing 1 mM EDTA, 0.5 mM, β-mercaptoethanol and PierceTM Protease Inhibitor Tablets, EDTA-free (lysis buffer). Samples were lysed by sonication (three bursts of 10 s) in an ice bath. The protein content was determined on the homogenates (crude lysates) according to the Lowry method. Crude lysates (10 μg/lane) were submitted to 10% SDS/PAGE and transferred to nitrocellulose membranes (Bio-Rad) by electroblotting. Membranes were blocked in 20 mM Na Phosphate buffer pH 7.4, 150 mM NaCl and 1% Tween20 (PBST) containing 5% non-fat dry milk for 1 h and incubated with primary antibodies (Supplementary Table S3) overnight at 4°C under gentle agitation. Immune-reactive signals were detected following incubation with the appropriate peroxidase-conjugated secondary antibody (Supplementary Table S3), using the ChemiDoc XRS apparatus and quantified using the Quantity One 4.6.1 software (Bio-Rad). Appropriate loading controls were used in order to minimize membrane stripping and when unfeasible probed on duplicate gels run in parallel.

### Hcast 3-25 cloning and transfection

Hcast 3-25 transcript (GenBank MK585065) was obtained by amplification of single stranded cDNA generated from 5 μg total RNA extracted from peripheral blood mononuclear cells. The amplicon was cloned into pEF1/V5-HisB mammalian expression Vector (Invitrogen) (the V5 epitope is at the C-terminus of the protein) using the forward primer Sn-BamHI 5′- AAGGATCCATGAATCCCACAGAAACCAAGG and reverse primer Asn-XbaI: 5′-AATCTAGACTTGAGTCATCTTTTGGCTTGGAA. PCR conditions were: a denaturation step for 1 min at 98°C; then 95°C for 30 s, 55°C for 30 s, and 72°C for 2 min, for 35 cycles. A 5 min extension step at 72°C was performed after the last cycle of PCR. One Shot TOP10 Chemically Competent *E. Coli* (Thermo Fischer Invitrogen) were transformed with pEF1/V5-HisB/hcast 3-25 construct, the ampicillin-resistant cells were selected, and the plasmid was purified using HiSpeed Plasmid Maxi Kit (Qiagen). The sequence of the cloned hcast 3-25 was confirmed by sequencing with CEQ 2000XL DNA analysis system (Beckman Coulter). Sub-confluent (∼60%) GBM3 cells were transfected with pEF1/V5-HisB/hcast 3-25 plasmid (2.5 μg) by Lipofectamine™ Stem Transfection Reagent (Invitrogen ThermoFisher Scientific) following the manufacturer’s instructions. GBM5 cells were transfected with pEF1/V5-HisB/hcast 3-25 plasmid (2.5 μg) by nucleofection with a Nucleofector TM II device using Amaxa Cell line Nucleofector Kit V (Lonza), using program T20. At 72 h post-transfection, cells were harvested and processed for RT-qPCR or WB analysis.

### Statistical analysis

All experiments were repeated at least three times and performed in triplicate. The results are expressed as mean ± SD. Comparisons were performed by unpaired, two-tailed Student’s t-test and statistical significance was set at *p* < 0.05, using GraphPad Prism version 7.0 (GraphPad Software, San Diego, California, United States).

## Results

### Gene expression profiling interactive analysis (GEPIA) shows higher expression of calp/cast system genes in GBM samples compared with normal samples

**FIGURE 2:**
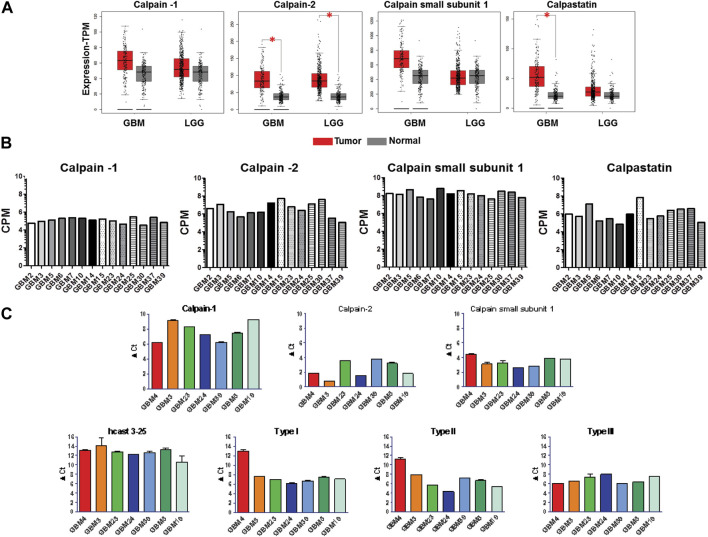
Expression of the calp/cast system components in normal brain, low grade glioma (LGG), glioblastoma (GBM) tissues and in patient-derived GBM stem cells. **(A)** Scattered-boxplots of differential transcript levels for calpain-1, calpain-2, calpain small subunit 1 and calpastatin determined by GEPIA database in 207 normal, 518 LGG and 163 GBM tissues. The signature score is calculated by mean value of TPM (transcripts per million) of each gene. *p < 0.05 (Student’s t-test). **(B)** Expression of the calp/cast system components in GBM stem cells isolated from 14 human tumors, evaluated by RNA-seq, and expressed as counts per million reads mapped (CPM). RNA-seq data are deposited at NCBI Geo data set (see Materials and Methods). **(C)** Total RNAs were isolated from 7 GBM stem cultures and RT-qPCR was performed with specific primers targeted to the indicated genes. mRNA expression was measured as ΔCt between the gene of interest and the matched RPLP0 used as housekeeping gene. Bars represent the mean ± SD from four experiments.

To explore the expression of the calp/cast system components in human GBM, we analyzed by GEPIA (http://gepia.cancer-pku.cn/) (Tang Z. et al., 2019) the mRNA expression level of the calp/cast axis in normal brain (207 samples), low-grade glioma (LGG, 518 cases) and high-grade glioma (GBM 163 cases) based on the Cancer Genome Atlas (TGCA) and Genotype-Tissue Expression (GTEx) database (Figure 2A). Results showed that Calp-2 and calpastatin mRNAs are significantly upregulated in GBM as compared to normal tissue, while Calp-1 and calpain small subunit 1 (CSS1) expression is higher in GBM than in normal brain without reaching statistical significance. In LGG, only Calp-2 expression is significantly higher than in normal samples. These results do not include known calpastatin subtypes and the new hcast3-25 form (Figure 1 for their exon/domain composition). Therefore, we performed preliminary mRNA analysis in established cell lines representative of low (Res186 and Res259) and high-grade (U87) gliomas by RT-qPCR (Supplementary Figure S1). When considering Cast as a whole (cast tot, without hcast 3-25), Res186 contained the lowest amounts of mRNA, while mRNA levels for hcast 3-25 are almost comparable. Type III, whose protein sequence is included in Type I and Type II (see Figure 1) and has the same 5′-UTR region of hcast 3-25 (Sparatore et al., 2019), is the only calpastatin isoform more expressed in U87 than in Res186 and Res259. Cycle threshold analysis (Supplementary Figure S1) evidenced that hcast 3-25 is the least represented transcript among the calp/cast system components in these cell lines.

### Patient-derived glioblastoma stem cell cultures (GSCs) highly express transcripts for calp/cast system components

Since established cancer cell lines scarcely recapitulate the phenotype/genotype of parental cells as well as the cell heterogeneity comprising the cancer stem phenotype of the original tumor *in vivo* (Lee et al., 2006), we focused our study on patient-derived GBM stem cells (GSCs). All the GSC cultures analyzed have been previously characterized for *in vivo* tumorigenicity, expression of tumor stem cell markers, multilineage differentiation ability (Griffero et al., 2009; Wurth et al., 2013), and grown *in vitro* in a stem-permissive serum-free medium. The transcriptome profile of the calp/cast system components (Calp-1, Calp-2, CSS1 and total Cast) was analyzed in a panel of 14 GSC cultures by RNA-seq (NCBI GEO Dataset #GSE179356) (Figure 2B). Overall, average gene expression across independent GSCs showed high levels of mRNA, particularly for CSS1, although reflecting the expected limited heterogeneity among distinct cultures.

### GSCs express different levels of mRNA for calpastatin isoforms and hcast 3-25

Considering that the multiple calpastatin transcripts were examined by RNAseq as a whole, we then analyzed the mRNA levels of the different Cast isoforms, including hcast 3-25, together with Calp-1, Calp-2 and CSS1, by RT-qPCR as a complementary technique to validate transcripts detected by RNA-seq (Figure 2C). To overcome potential biases due to GBM heterogeneity, we tested seven independent patient-derived GSC cultures (GBM3-4-5-19-23-24-39), selected considering the ease of *in vitro* maintenance (i.e., growth rate) to ensure technical feasibility and a good level of replicability (see Supplementary Table S1). All the GSCs express mRNA levels for the catalytic subunit of Calp-1 lower than those for Calp-2, and the transcription of CSS1 (the regulatory subunit of both Calp-1 and -2) within the same GBM culture, seems unrelated to the mRNA levels of the catalytic subunits. Except for GBM4 that is characterized by lower amounts of Type I and II calpastatins, the other cultures showed comparable amounts of Type I, II and III (ranging from 5-8 ΔCt).

Instead, the mRNA levels for hcast 3-25 were lower than those of the other Cast isoforms and, despite hcast 3-25 and Type III share the same 5′-UTR region (Sparatore et al., 2019), they displayed the greatest variability among the different GSCs as well as in the reciprocal ratios of the two transcripts within the same culture (compare hcast 3-25 and Type III graphs in Figure 2C). In addition, it is worth recalling that the “inhibitory” full-length Casts only differ in the N-terminal non-inhibitory domain (Figure 1) and potentially exert identical inhibitory activity. The occurrence of additional Cast forms resulting from alternative splicing in the L-domain, suggests that calpastatin does not behave exclusively as calpain inhibitor but plays roles still unknown, not related to inhibition.

### GSCs show comparable protein amounts of calpains, CSS1 and total calpastatin but specific ratios of native/autoproteolyzed Calp-1

**FIGURE 3:**
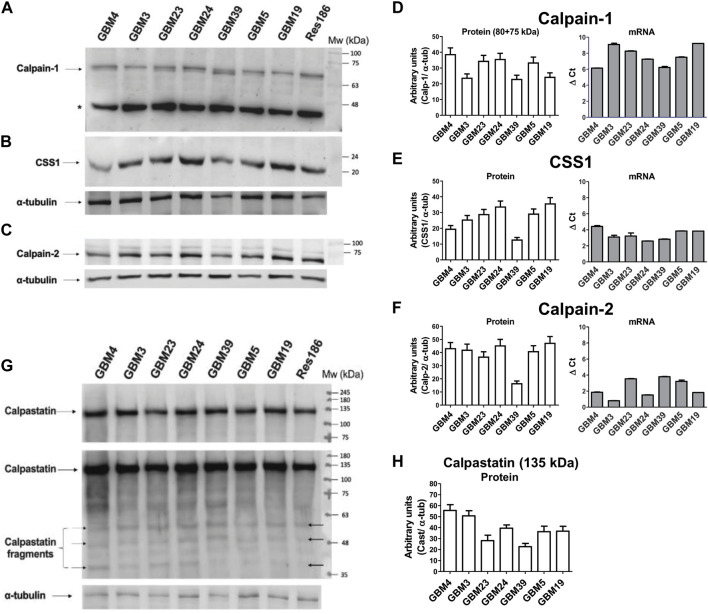
Levels of protein and mRNA for calpain-1, calpain-2, CSS1 and calpastatin in GBM stem cells. Proteins from crude lysates (10 μg) prepared from the indicated GBM cultures were separated by SDS/PAGE (10%) and detected by immunoblotting. Upper panel: Representative WB of **(A)** calpain-1, **(B)** CSS1 and **(C)** calpain-2. α-tubulin was detected following stripping of calp-1 and CSS1 WB membrane, while Calp-2 was run in parallel on another gel and immunodetected together with α-tubulin. Res 186 cells are reported as running condition control. (*) Non-specific immune-reactive signal corresponding to Calp-1 digestion product (∼46 kDa) (De Tullio et al., 2018). Histograms: **(D)** Calp-1, **(E)** CSS1 and **(F)** Calp-2. Bars represent protein levels (white bars) calculated by densitometry analysis normalized to α-tubulin used as loading control. mRNA levels (grey bars, see Figure 2C) of **(D)** Calp-1, **(E)** CSS1 and **(F)** Calp-2 were measured as ΔCt between the gene of interest and the matched RPLP0 used as housekeeping gene. Bars represent the mean ± SD from four experiments. Lower panel: **(G)** Representative WB of Cast. The second image shows a longer exposure of the same blot in which several calpastatin fragments are faintly visible (black arrows). Res 186 cells are also reported. **(H)** Bars are the mean values ± SD from 3 replicates and represent protein quantification of native Cast (135 kDa) determined by densitometry analysis normalized to α-tubulin used as loading control.

We then evaluated the protein content of the calp/cast system components in the seven GSC cultures by Western blotting. Each culture showed a unique content of native (80 kDa) and autoproteolyzed/activated (75-kDa) Calp-1 forms (Figure 3A). Specifically, GBM3 and GBM19 showed only the native form; GBM4, GBM23, GBM24, and GBM5, regardless the total amount of Calp-1, contained also the 75-kDa species, whereas in GBM39 activated Calp-1 overcame the native form. Res 186 were loaded on gels as size-separation and running condition control.

Conversely, Calp-2 species resulting from autoproteolysis were undetectable (Figure 3C), and except for GBM39 that contained the lowest Calp-2 protein amount among the GBMs examined (Figure 3F), the other cultures displayed comparable levels of Calp-2 catalytic subunit.

The protein content of CSS1 (normally associated to both catalytic subunits in a ratio 1:1) did not seem related to the amounts of either Calp-1 or Calp-2 catalytic subunits. In fact, GBM4 and GBM39 expressed low amounts of CSS1 protein, but the latter also contained low levels of both Calp-1 and Calp-2 catalytic subunits (Figures 3B–E). By comparing the protein with the corresponding mRNA levels of Calp-1, Calp-2 and CSS1 (Figures 3D–F) it can be observed that lower ΔCt does not correspond to higher amounts of protein.

Finally, we measured the amount of Cast (Figures 3G–H) using an antibody to the fourth inhibitory domain, which neither distinguishes between the various Cast forms (see Figure 1) nor recognizes hcast 3-25 that lacks the inhibitory domains. Cast protein is mainly present in the native high Mw form (about 135-kDa, Figure 3G), and when the same WB was analyzed after a longer exposure (60 s) (Figure 3G, middle panel), several immune-reactive bands corresponding to Cast species deriving from calpain-dependent proteolysis were detected. Overall, these data show that all the patient-derived GSC cultures contain similar protein amounts of Calp-1, Calp-2, CSS1 and total cast (excluding hcast 3-25) but, as previously observed in human meningioma tissues (De Tullio, et al., 2018), each culture is characterized by a specific ratio of native vs. autoproteolyzed Calp-1 species reflecting the heterogeneity of the original GBM.

In the series of GSCs analyzed, the calp/cast system appears more activated in GBM39 because these cells predominantly contain the 75-kDa Calp-1 form and lower amounts of Calp-2, CSS1 and Cast (Figure 3). However, since Cast behaves at the same time as substrate and inhibitor of calpain, its digestion cannot be a marker of the actual activity of the calp/cast system in GSC lysates. Thus, we analyzed the cleavage of α-II spectrin, a calpain target yielding specific 150-kDa and 145-kDa fragments in the CNS (Czogalla and Sikorski, 2005). We found that GSC cultures, except GBM4 and GBM39, contained comparable amounts of native α-II spectrin (Supplementary Figure S2) and the digested forms (150 + 145-kDa) accounted for about 90% of the total protein in GBM4 and GBM39% vs. 70% of the other cultures.

Increased degradation of α-II spectrin did not correspond to higher transcription of hcast 3-25. GBM4 and GBM39, showing the highest percentage of spectrin digestion (Supplementary Figure S2) displayed hcast 3-25 mRNA levels lower than GBM23 and GBM19 which showed similar percentage of α-II spectrin digestion and similar amounts of Calp-2 and CSS1, as well as the prevalence of native Calp-1 (see Figure 3). Essentially, we found no correlation between hcast 3-25 expression and α-II spectrin proteolysis in the GBMs analyzed.

### The transcription of all the calp/cast system components, except hcast 3-25, is increased in GBM differentiated cells

Ca^2+^ homeostasis and calpains are involved in cancer stem cell functions, differentiation, plasticity and capacity for adaptation, fueling tumor heterogeneity. Hence, we explored the modulation of the calp/cast system in four GSC cultures (GBM3, GBM23, GBM5 and GBM19) showing different amounts of hcast 3-25 mRNA (see Figure 2C), optimal *in vitro* growth rate and handling following differentiation in a medium containing 10% FBS for at least 15 days. Growth factor (EGF, bFGF) withdrawal from culture media results in spontaneous differentiation of GSCs mainly towards astrocyte-like phenotype and reduced proliferation (Griffero et al., 2009; Carra et al., 2013; Wurth et al., 2013; Gritti et al., 2014). The differentiated cells (GBM diff) adhere to the substrate without Matrigel coating and acquire an elongated shape and branching, compatible with a glial-like morphology (Supplementary Figure S3). To confirm that differentiation of GBM stem cells occurred, GFAP (glial fibrillary acidic protein), a primary intermediate filament of mature astrocyte, and SOX2 (Sry-box transcription factor 2), a transcription factor enriched in populations with stem cell properties, were analyzed by WB. GFAP, except in GBM5, was markedly increased in GBM3, GBM23 and GBM19 differentiated cells compared to the stem counterpart, while SOX2 decreased in all the differentiated cultures (Supplementary Figure S4).

**FIGURE 4:**
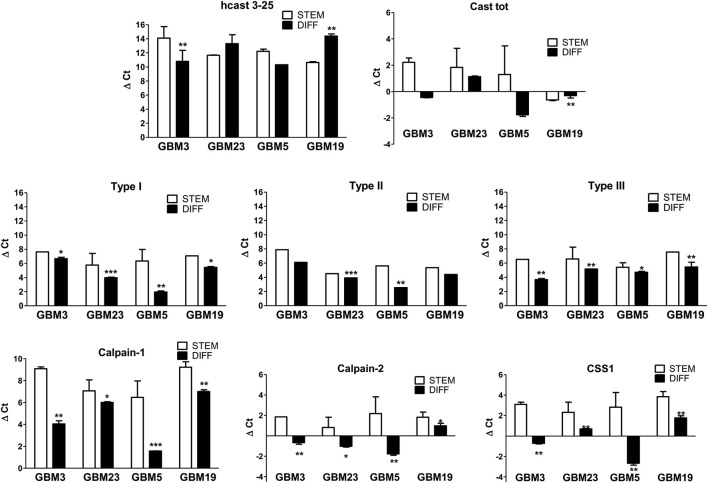
Comparison of mRNA expression pattern of the calp/cast system components between stem and differentiated GBM cultures. Total RNAs were isolated from stem GBM3, GBM23, GBM5, GBM19 cells and after differentiation. RT-qPCR was performed with specific primers targeted to the indicated genes (see Figure 2C). mRNA expression was measured as ΔCt between the gene of interest and the matched RPLP0 used as housekeeping gene in the stem (STEM, white bars) and differentiated (DIFF, black bars) conditions. Bars represent the mean ± SD from four experiments. *p < 0.05, **p < 0.01 and ***p < 0.001 vs. the respective GBM stem culture by unpaired, two-tailed t-test.

After differentiation, the mRNA levels for hcast 3-25 (Figure 4) significantly increased in GBM3 and decreased in GBM19 while no significant differences were observed in GBM5 and GBM23 (Supplementary Table S4 for details about fold changes), and Type III transcription was upregulated in all four GBM cultures regardless of the expression of hcast 3-25. In fact, the highest increase in Type III mRNA was observed in GBM3 diff, matching the highest upregulation of hcast 3-25 mRNA, while GBM19 diff, showed the largest decrease in hcast 3-25 transcription, interestingly, despite having the same four inhibitory units, the expression of the classical Casts (Type I, II and III) increased upon differentiation in all the four differentiated cultures although varying among GBMs (Figure 4).

Total Cast mRNA levels (measured using two primers in the second inhibitory domain, thus excluding hcast 3-25) were enhanced after differentiation in all cultures, regardless of the initial amount of the mRNAs in the stem state (see Supplementary Table S4).

The transcripts for Calp-1, Calp-2 and CSS1 variably increased in differentiated GBM cells compared with their stem counterpart. Calp-1 mRNA was strongly upregulated (ranging from +469-fold in GBM19 to 6057-fold in GBM5), while Calp-2 expression increased to a lesser extent. Finally, CSS1 mRNA was more upregulated in GBM3 diff +1384) and GBM5 diff (+9148), that showed concomitantly an increase in hcast 3-25 mRNA, than in GBM19 diff (+427-fold) and GBM23 diff (+709-fold) characterized by a decrease in hcast 3-25 transcription (Figure 4; Supplementary Table S4).

Collectively, transcription of all the calp/cast system components, except hcast 3-25, increased following differentiation.

### The protein turnover and activity of the calp/cast system components are increased in GBM differentiated cells

**FIGURE 5:**
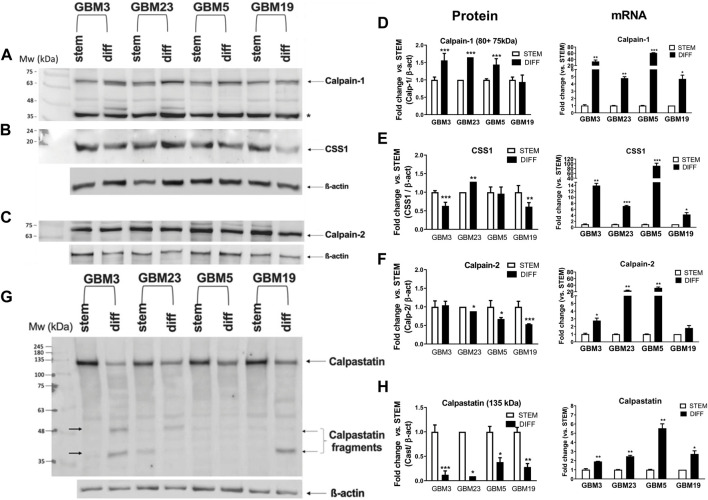
Comparison of protein and mRNA levels of calpain-1, calpain-2, CSS1 and calpastatin between stem and differentiated GBM cultures. Proteins from crude lysates (10 μg) prepared from GBM3, GBM23, GBM5, and GBM19 cultures, before (STEM) and after differentiation (DIFF), were separated by SDS/PAGE (10%) and detected by immunoblotting. Upper panel: Representative WB of **(A)** Calp-1, **(B)** CSS1, and **(C)** Calp-2. Densitometry analysis for protein levels were normalized to β-actin that was detected following stripping of calp-1 and CSS1 WB membrane. Calp-2 and β-actin were detected on a membrane from another gel run in parallel. (*): non-specific immune-reactive signal corresponding to Calp-1 digestion product (∼46 kDa) (De Tullio et al., 2018). Histograms: protein and mRNA levels of **(D)** Calp-1, **(E)** CSS1 and **(F)** Calp-2 in GBM stem (white bars) and GBM diff (black bars) cultures. Bars represent protein amounts calculated by densitometry analysis normalized to β-actin used as loading control. Protein and mRNA levels are expressed as fold change expression vs. the respective GBM stem culture and RPLP0 was used as housekeeping gene. Lower panel: **(G)** Representative WB of Cast. Calpastatin fragments are indicated by black arrows. **(H)** Bars represent protein quantification of native Cast (135 kDa) by densitometry analysis in the indicated stem (white) and differentiated (black) GBM cultures. Protein levels were normalized to β-actin and data are expressed as fold change vs. the matching stem GBM culture. β-actin was detected both following stripping and in parallel on another WB membrane from gels run in parallel, because its electrophoretic mobility is close to the calpastatin fragments generated during calpain-mediated digestion. mRNA levels are expressed as fold change vs. STEM. Data are reported as means ± SD (n = 3). *p < 0.05, **p < 0.01 and ***p < 0.001 vs. the respective GBM stem culture by unpaired, two-tailed t-test.

We then measured the protein amounts of the calp/cast system components following GSC differentiation. By comparing their levels in the stem and differentiated state (Figure 5), we observed that: 1) All the four cultures displayed a significant increase in Calp-1 protein (80 + 75-kDa) and both GBM3 and GBM23 contained an additional fragment (∼46-kDa) deriving from autoproteolysis (Cong et al., 1989). Calp-1 protein upregulation parallels with increased transcription in these GBMs (Figure 5D); 2) Calp-2 protein was weakly decreased after differentiation despite a marked mRNA increase (Figure 5F); 3) CSS1 protein, except for GBM23, was downregulated (Figure 5E) while transcription increased (most notably in differentiated GBM3 and GBM5).

These data suggest that during differentiation an increased turnover of these proteins occurs.

Concerning calpastatin (Figures 5G, H), the amount of native protein (135 kDa) was significantly reduced upon differentiation in all four cultures despite the concomitant increase in transcription 4. Except for GBM5, the other GBMs showed additional low Mr bands corresponding to Cast fragments retaining the immune-reactive fourth inhibitory domain (see arrowed bands in Figure 5G).

All these observations support the hypothesis that the calp/cast system is more activated in differentiated GBM cells than in the stem counterpart. Indeed, the decrease in native Cast protein indicates a sustained calpain-dependent “consumption” of the inhibitor, and the presence of free inhibitory units suggests that calpain activity is acting through limited proteolysis.

The increased activation of the calp/cast system did not lead to increased cleavage of α-II spectrin in differentiated GBM cells (Supplementary Figure S5). Although each stem and differentiated culture shows a specific amount of native and digested α-II spectrin species, they did not exhibit changes in the ratio digested/total α-II spectrin, and similarly to the seven stem GSCs (Supplementary Figure S2), such a ratio lies in a range between 70%–80%.

### Overexpression of hcast 3-25-V5 in GBM3 and GBM5 stem cultures results in an increased activity of calpains on their targets without affecting the transcription of the calp/cast system components

**FIGURE 6:**
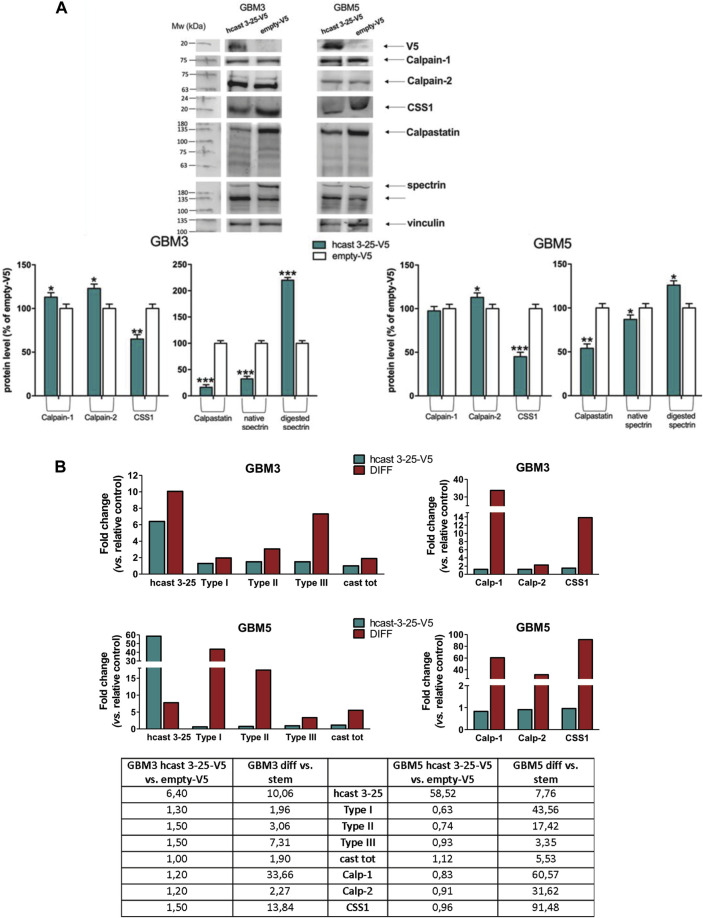
Transient transfection of hcast 3-25-V5 in GBM3 and GBM5 stem cells: expression profiles of the calp/cast system components. GBM3 and GBM5 cells were transfected with hcast 3-25-V5 or empty-V5 and after 72 h cells were collected for preparing protein crude lysates [immunoblotting, panel **(A)**] and total mRNA [RT-qPCR, panel **(B)**]. **(A)** Representative WB of the indicated proteins detected following transfection with hcast 3-25-V5 or empty-V5. Vinculin was used for protein loading control and normalization. Histograms: Bars are the mean values of densitometry analyses ±SD from 3 replicates and data are expressed as percentage of control (empty-V5). **(B)** mRNA levels of the indicated genes were evaluated in GBM3 and GBM5 cells transfected with hcast 3-25-V5, or after differentiation, by RT-qPCR. Fold changes, reported in histograms and table, were calculated considering GBM3- (and GBM5-) hcast 3-25-V5 vs*.* matching empty-V5 cells and GBM3 (and GBM5) diff vs*.* their stem counterpart (see also Figure 4; Supplementary Table S4). RPLP0 was used as housekeeping gene. *p < 0.05, **p < 0.01 and ***p < 0.001 relative to empty-V5 cells by unpaired, two-tailed t-test.

Given the above findings, we performed transient overexpression of hcast 3-25 in the two GSC cultures (GBM3 and GBM5, see Figure 4) showing increased hcast 3-25 transcription following differentiation. GBM3 stem cells were transfected with hcast 3-25-V5 (the V5 tag is at the C-terminus) or empty-V5 plasmid using cationic-lipid transfection reagent, a method less successful in efficiently transfecting GBM5 for which we used an optimized protocol to transfer plasmid directly into the nucleus (see Material and Methods). Cells were harvested 72 h post-transfection and analyzed for relevant mRNA and protein expression (Figure 6). Even if levels of mRNA for hcast 3-25 markedly differed (+6.40-fold in GBM3 vs*.* +58.52-fold in GBM5 compared to respective empty-V5, Table in Figure 6B), the effects on proteins (Figure 6A) and mRNAs (Figure 6B) of the calp/cast system components were evident and almost superimposable in both GBM3 and GBM5. The protein levels of Calp-1 and Calp-2 catalytic subunits (except Calp-1 in GBM5) were significantly increased in hcast 3-25-V5 compared to empty-V5; both CSS1 and Cast were partially digested in hcast 3-25-V5 transfected cells indicating that in the presence of increased amounts of hcast 3-25-V5, calpains are more active on their targets, in both GSC cultures. The increased activity of calpains was also confirmed by the accumulation of the 135-kDa α-II spectrin fragment in GBM3-and GBM5-hcast 3-25-V5 (Figure 6A).

Transfection of hcast 3-25 did not affect the transcription of the calp/cast system components since there were no significant differences between GBM3- and GBM5-hcast 3-25-V5 and the empty-V5-trasfected counterparts (Table in Figure 6B, first and fourth columns). The mRNA levels of these genes in transfected GBM stem were then compared with those of serum-induced differentiated cultures (Figure 6B, second and fifth columns in the Table, see also Supplementary Table S4). Except for hcast 3-25 expression that was 6.40-fold increased in transfected vs. +7.40-fold in differentiated GBM3 and +58.52-fold in hcast 3-25-V5-GBM5 compared to +7.76-fold in GBM5 diff, all the other transcripts resulted almost unchanged in both transfected cultures. Transient overexpression of hcast 3-25 did not induce even partially the increases in the transcription of the calp/cast system components observed after 15 days of differentiation but analogously to serum-differentiated cells, resulted in increased activity of calpains finally yielding to the limited digestion of CSS1, Cast and α-II spectrin.

**FIGURE 7:**
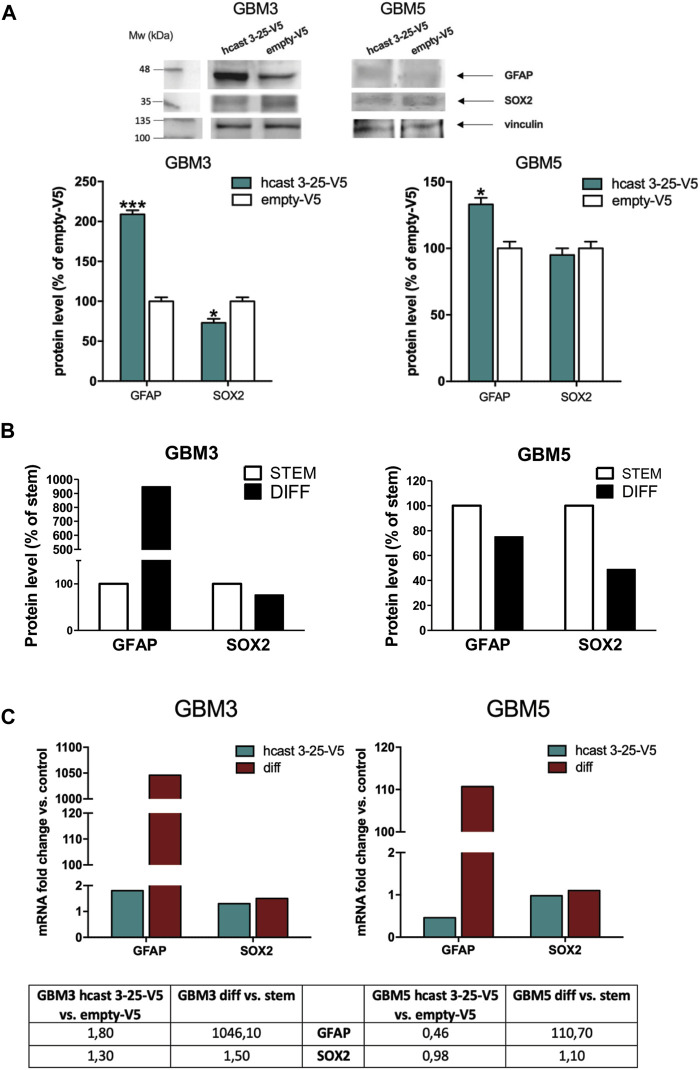
Transient transfection of hcast 3-25-V5 in GBM3 and GBM5 stem cells: expression profiles of GFAP and SOX2. **(A)** Representative WB of GFAP and SOX2 detected following transfection with hcast 3-25-V5 or empty-V5. Vinculin was used for protein loading control and normalization (immunodetected on the same WB membrane). Bars are the mean values of densitometry analyses ±SD from 3 replicates and data are expressed as percentage of control (empty-V5). **(B)** Bars represent protein levels of GFAP and SOX2 (see Supplementary Figure S4) in differentiated GBM3 and GBM5 cultures (black bars) expressed as percentage of matching stem cells (white bars). **(C)** mRNA levels of GFAP and SOX2 were evaluated in GBM3 and GBM5 following transfection with hcast 3-25-V5, by RT-qPCR. Fold changes were calculated as in Figure 6B considering their matching controls (GBM3- and GBM5-empty-V5 or GBM3 and GBM5 stem cells). Values are normalized vs. RPLP0, used as housekeeping gene. *p < 0.05, **p < 0.01 and ***p < 0.001 relative to empty-V5 cells by unpaired, two-tailed t-test.

We next investigated the protein amounts of the astroglial marker GFAP and the stem cell transcription factor SOX2 in GBM3-and GBM5-hcast 3-25-V5 (Figure 7). GBM3-hcast 3-25-V5 expressed significantly higher GFAP (+110%) and decreased SOX2 (−27%) protein amounts than GBM3 empty-V5, as was observed following serum-differentiation (Figure 7B; Supplementary Figure S4). In hcast 3-25-V5 transfected GBM5, GFAP protein was significantly increased (+33%) while SOX2 did not change (Figure 7A). Interestingly, both stem and differentiated GBM5 cells are characterized by negligible protein amounts of GFAP (Figure 7B; Supplementary Figure S4). Overexpression of hcast 3-25 does not affect the mRNA levels for GFAP and SOX2 (Figure 7C), but more likely mimics the calp-cast proteomic profile and activity of the differentiated state.

## Discussion

The modulation of the calp/cast system correlates with multiple physiological and pathological processes, including cancer. While the role of calpains is mainly ascribed to the proteolytic cleavage of selected targets, calpastatin biological function is intrinsically related to calpain inhibition. Indeed, very little is known about the calpastatin regulatory/non-inhibitory domain localized upstream of the inhibitory units and undergoing alternative splicing. Besides full-length calpastatins responsible for calpain inhibition, we identified a new calpastatin named hcast 3-25 in human CNS, devoid of the four repetitive inhibitory units and containing the N- (exon 1u-3) and C-terminal (exons 25-29) of calpastatin Type III (Sparatore et al., 2019). The challenge of studying hcast 3-25 *ex vivo* is the impossibility of measuring its activity on calpain, and the lack of a specific antibody against the protein. Furthermore, since calpastatin undergoes calpain-dependent digestion (De Tullio et al., 2000; Stifanese et al., 2010), it is impossible to distinguish among native low Mr Casts (i.e., hcast 3-25) and the various fragments containing the identical C-terminal end, common to all calpastatins.

In human GBM, the transcriptomic data elaborated from the TCGA and GTEx gene expression datasets show that calpain and calpastatin are upregulated in GBM tissues compared to normal brain tissue, likely suggesting a role of the calp/cast system in tumor development and aggressiveness. Recent proteomic data (Stillger et al., 2023), although obtained from a limited series of GBMs, indicate upregulation of Calp-2 and, to a lesser extent, of Calp-1, compared to adjacent brain tissue.

Interestingly, Calp-2 gene expression was also significantly higher in LGGs than in normal tissue, while Calp-1, CSS1, and Cast levels were comparable to normal samples and lower than in GBM. LGGs include a heterogeneous group of tumors generally characterized by well-differentiated cells, low growth rate and invasiveness, less aggressive behavior, and better prognosis than GBM. Therefore, we first analyzed the mRNA amounts of the different Calp and Cast isoforms in classical preclinical models of LGG (Res186, Res259) and GBM (U87) cell lines. Overall, calpains and calpastatins were upregulated in LGG cell lines, poorly mirroring the tumor sample expression profile. Glioma cell lines, derived from patients’ samples but grown indefinitely in serum-enriched media, scarcely recapitulate the original tumors’ *in vivo* genotypic and phenotypic features, and lose the tumorigenic cancer stem cell population (Lee et al., 2006). Thus, we used low-passage serum-free stem cell cultures derived from primary GBMs as a more reliable model for preclinical studies. We analyzed 14 patient-derived GSC cultures by RNA-seq, confirming high transcription levels of the calp/cast system components, and then we focused on hcast 3-25 mRNA in 7 of the 14 cultures.

While Calp-1, Calp-2 and CSS1 mRNAs were higher in cell lines than in GSC cultures, the Cast isoforms were variably expressed among the individual GSC cultures, indicating that GSCs retain patient-specific expression. These observations are consistent with a differential usage of the three promoters upstream of exons 1xa, 1xb, and 1u, each one containing transcription factor-binding motifs sensible to a variety of stimuli (Sensky et al., 2006; Masilamani et al., 2022). Therefore, transcription of the different Cast variants changes according to stimuli received during GBM growth and development, producing molecules with inhibitory activity that differ in the non-inhibitory domain. Calpastatin can also undergo internal splicing in the L-domain by removing single exons. Such a process generates other full-length isoforms starting with the same Met but having a peculiar set of exons in the L-domain (Sensky et al., 2006; De Tullio et al., 2007; Sparatore et al., 2019).

In the case of hcast 3-25, the internal splicing occurs to Type III and generates a variant lacking the inhibitory units but containing exon 3. Exon 3 is prevalently skipped in porcine and human full-length Casts (Parr et al., 2004; Sparatore et al., 2019), whereas it is differentially transcribed in beef muscle (Motter et al., 2021). Storr et al. found an association between Cast-containing exon 3 and lymphovascular invasion in breast cancer (Storr et al., 2011b). Interestingly, hcast 3-25 and Type III show the most significant fluctuations among the seven GSCs, and their reciprocal ratio within the same sample was unrelated. Hcast 3-25 is the least represented variant among the different Casts isoforms, suggesting that, due to the lack of inhibitory units, it may play a decisive role in supporting the activity of these proteases. Indeed, calpains exert their function despite cells containing calpastatin activity exceeding the activity of the two calpains together (Goll et al., 2003). In GSCs, hcast 3-25 expression correlated neither with the mRNA levels of the other Cast isoforms nor with the transcription of calpains.

Based on previous experiments using recombinant hcast 3-25 (Sparatore et al., 2019), we hypothesized that the GSCs showing top levels of hcast 3-25 mRNA (GBM19 and GBM23) could be characterized by increased activation/activity of the calp/cast system. Due to colocalization of calpains and the inhibitory effect of calpastatins, the activity of the two proteases cannot be directly measured in crude lysates, thus we used the autoproteolyzed forms of the catalytic subunits together with CSS1 digestion and Cast low Mr species, as markers of calpain activation (Stifanese et al., 2010), and α-II spectrin, a specific calpain target, as a marker of calpain activity (Czogalla and Sikorski, 2005; Higuchi et al., 2012).

Several Authors demonstrated that CSS1 modulates the proliferation and metastasization of different types of human cancer cells (Li et al., 2014; Cai et al., 2016; Cheng et al., 2018), including gliomas (Cai et al., 2014; Cai et al., 2016). A recent meta-analysis reports that overexpression of CSS1 protein is closely related to worse outcomes and more aggressive tumor features in cancer patients (Tang S. et al., 2019). We observed that all the GSC cultures show similar levels of both CSS1 mRNA and protein (except GBM39 that contained very low amounts of protein). Notably, all the cultures were obtained from high grade gliomas.

It has been widely demonstrated that overexpression of the calpastatin inhibitory unit helps to relieve the effects of excessive calpain activity (Aluja et al., 2022), also in the CNS (Schoch et al., 2012; Diepenbroek et al., 2014; Yu et al., 2020). Since calpastatin behaves simultaneously as a calpain inhibitor and substrate, the presence of discrete Cast fragments indicates that the calp/cast system is activated. Hcast 3-25, by protecting the activated protease from degradation, might support the limited digestion of calpastatin (producing free inhibitory domains) and other calpain targets.

In this context, evaluation of calpain-specific α-II spectrin 150 and 145 kDa breakdown products in the CNS (Czogalla and Sikorski, 2005; Higuchi et al., 2012) allowed us to measure the actual calpain activity in each GSC culture at the moment of processing. Indeed, the presence of Cast fragments indicates the activation of the calp/cast system. However, α-II spectrin digestion might occur only if calpastatin inhibitory units do not occupy the calpain catalytic site.

Thereby, GBM4 and GMB39, in which we observed the highest cleavage of α-II spectrin, contained the lowest CSS1 and/or Cast protein amounts (GBM4 contain high levels of native Cast but the digested forms are also present, see Figure 3G). As expected, due to the different genotype of the patient-derived cultures, each GSC displays a different activation pattern of the calp/cast system evaluated as the content of native and activated Calp-1 and Calp-2 forms. Detection of similar amounts of CSS1 and the prevalence of native Cast revealed that in these cultures, the calp/cast system seems to act in basal conditions (Stifanese et al., 2010).

Overall, no correlation between the extent of calpain activation/activity and the transcription of hcast 3-25 was detected, but data confirm that the effective cleavage of calpain targets results from a balanced combination of the various calpain and calpastatin isoforms and species (including “the invisible” hcast 3-25) available in each GSC culture, and not from their absolute amount.

In light of these observations, we rescheduled our strategy to obtain information about hcast 3-25 by selecting four GBM stem cultures showing low-medium-high transcription of hcast 3-25 (see Figure 2C) and explored the behavior of the calp/cast system following their differentiation. Indeed, the ability to differentiate (“non-GSCs”) is an expected although not essential property of GSCs. Moreover, the contribution of the stemness-to-differentiation axis to cellular features in GBM support the modulation of GSC tumor-propagating potential (Piccirillo et al., 2006; Stricker et al., 2013).

Differentiation involves calpain activity also in the CNS (Baudry and Bi, 2016; Shu et al., 2018; Nian and Ma, 2021) and transcription of cast Type III is upregulated during migration and differentiation of high-grade gliomas (Vo et al., 2019a). We found that except hcast 3-25, transcription of all the other components of the calp/cast system increased following differentiation. Concerning cast Type III, our observations are in agreement with the data obtained by Vo et al. (2019a), but we also found upregulation of Type I and Type II. Significantly, considering the transcription of the full-length Casts containing inhibitory domains (cast tot) in differentiated GBM cells, we observed that it increases up to 3-fold compared to the stem condition (see Figure 4), a minimal change compared to the upregulation of Calp-1 (up to 40-fold) and Calp-2 catalytic subunits. These data, combined with those obtained by WB analysis, indicate that differentiated cells compared to stem counterparts show: 1) increased Calp-1 activation, as demonstrated not only by the presence of the 75-kDa form but also by detection of low Mr inactive fragments (Ono and Sorimachi, 2012). The increased amounts of 80-kDa species indicate that Calp-1 consumption is efficiently counterbalanced by synthesis; 2) increased Calp-2 activation. Although autoproteolyzed Calp-2 was undetectable, the native protein decreased despite upregulated synthesis (Figure 5), suggesting that during differentiation, Calp-2 activation/degradation is faster than synthesis of new molecules (Baudry and Bi, 2016; Shu et al., 2018; Nian and Ma, 2021); 3) increased CSS1 mRNA level not matching with the corresponding protein amount. The lack of correspondence may be explained by several observations indicating that CSS1, in addition of being the regulatory subunit of calpain, is also related to tumor malignancy. Indeed, CSS1 plays a role in regulating the migration of several types of cancer cells (Li et al., 2014; Chen et al., 2019), including nasopharyngeal carcinomas cells infected by Epstein-Barr virus (Zheng et al., 2020). CSS1 is also targeted by miR-124, and its downregulation or miR-124 mimics suppress the migration and invasion of glioma cells *in vitro* (Cai et al., 2016). It is still unclear whether the CSS1 molecules acting in these tumors come from the pool that dissociated from calpain catalytic subunit following Ca^2+^ exposure or have been produced in excess and act independently from the calp/cast system; 4) increased Cast digestion. Cast has been cleaved by calpain since in all the four GBM differentiated cultures the amount of native protein is decreased compared to the stem condition. Since we used an anti-calpastatin antibody recognizing the fourth inhibitory domain, the low Mr fragments in GBM diff (Figure 5G) play an active role in maintaining the regulation of the calp/cast system. Indeed, even though both calpains are more activated following differentiation, the percentage of digested α-II spectrin is approximately comparable to that observed in the GBM stem (Supplementary Figure S5).

Although we have analyzed only four distinct GBM cultures, and the heterogeneity of the individual GBM may influence results, our data suggest that during differentiation calpains are differently activated and the calp/cast system seems to act in a physiological controlled condition. While differentiation induces upregulation of all the calp/cast system components, hcast 3-25 mRNA increases or decreases with respect to the stem state, depending on the GBM culture considered, suggesting that it plays a role finely tuned not exclusively related with differentiation.

We then induced overexpression of hcast 3-25 by transfecting hcast 3-25-V5 in GBM3 and GBM5 stem cells, the two GSC cultures that showed upregulation of hcast 3-25 following differentiation (Figure 4). Our observations demonstrate that hcast 3-25-V5 effectively competes with the endogenous calpastatins for the binding to calpain thus maintaining the protease active on its targets, *ex vivo*.

Transfection of primary and stem cells is often challenging, and we found it difficult to optimize methods for hard-to-transfect GBM3 and GBM5 stem cells, testing different commercially available transfection reagents. Efficient transfection of GBM3 was achieved with Lipofectamine™ Stem reagent, and Nucleofection technology was used for GBM5. Although the differences in hcast 3-25-V5 transcript levels between the two GBM cultures (hcast 3-25-V5 mRNA was increased 6.4-fold in GBM3 and 58.52-fold in GBM5 compared to respective empty-V5 controls), transient overexpression of hcast 3-25-V5 did not affect the transcription of the other calp/cast system components, GFAP and SOX2, as observed in 15-days-serum differentiated GBM3 and GBM5 cells. Significantly, in both transfected cultures, hcast 3-25-V5 overexpression affected the corresponding proteins since all the calpain targets analyzed were digested independently from the amount of hcast 3-25-V5 produced. Hcast 3-25 overexpression was also associated with significantly increased protein amounts of the astroglial marker GFAP and decreased levels of the stem marker SOX2. In this context, although GBM5 expressed the highest amount of hcast 3-25-V5 protein, we observed the same trend in both transfected stem cultures. As predictable, changes in GFAP and SOX2 mRNA and protein expression differ between 72 h-transfected cultures and 15-day FBS-differentiated counterparts in terms of absolute values since FBS contains a pool of various growth factors and components, which can modulate multiple molecular and cellular mechanisms underlying the differentiation process. Thus, it is possible to explain why a more efficient transfection of hcast 3-25 does not lead to increased cleavage of calpain targets and a more robust modulation of the stem and differentiation markers. Considering that upon FBS-induced differentiation, we observed that two GBM cultures out of four are characterized by upregulation of hcast 3-25, and the other two show downregulation (see Figure 4), we might hypothesize that this “anomalous” calpastatin is in some way involved in the differentiation of GSCs.

Finally, it is helpful to underline that cancer stem cells are not clonal entities, defined by distinct transcriptomic signatures and functional properties, but rather a cellular state driven by environmental conditions. In particular, stem cell-associated phenotypic heterogeneity is a feature of GBM; therefore *in vitro* inducible transition from a more undifferentiated to a differentiated state only mimics a complex *in vivo* dynamic process (Cui et al., 2023).

We suggest that hcast 3-25, preserving instead of inhibiting calpain activity, provides an additional regulatory mechanism of the calp/cast system acting in combination with the full-length calpastatins. Because it maintains calpain activity, hcast 3-25 could be important for prolonging calpain action in the presence of plenty of inhibitory units deriving from calpain-dependent cleavage. Interestingly, we previously demonstrated that the L-domain also plays a crucial role in regulating calpastatin’s inhibitory efficiency by determining the formation of complexes in which calpain can be fully inhibited or fully active (De Tullio et al., 2014).

This aspect is worth further investigation since calpastatin availability for calpain inhibition is related also to post-translational modifications, such as phosphorylation in the regulatory domain involved in calpastatin diffusion/aggregation (De Tullio et al., 2009). A recent paper reports that PKA-mediated phosphorylation of Ser 133 in human calpastatin domain I (in exon 8) enhances the inhibition of calpain and, consequently, postmortem calpain-dependent meat tenderization results decreased (Bai et al., 2023). The absence of inhibitory domains and the ability to extend the duration of calpain activity makes hcast 3-25 a promising tool for upregulating calpain in postmortem conditions.

In conclusion, a deeper understanding of the hcast 3-25 transcriptional regulation during GBM cell differentiation, a process leading to impairment of proliferation rate and invasiveness of GBM tumor cells could be critical for identifying new patient-specific GBM stem cell vulnerability and driving GBM stem cell fate. Due to the high undifferentiated profile of GBM, differentiation therapy has been proposed as a promising strategy for modulating GBM aggressiveness (Jin et al., 2017; Stevanovic et al., 2021; Liu et al., 2023). Thus, hcast 3-25 may represent an additional regulator of GBM stem cell phenotype. Therefore, deciphering novel molecular pathways such as the calp/cast system, sustaining GBM cell stemness, could prospectively serve as a tool for prognostic and/or therapeutic purposes, targeting therapy-resistant GBM stem cells, the population which fuels GBM initiation and recurrence.

**FIGURE 8:**
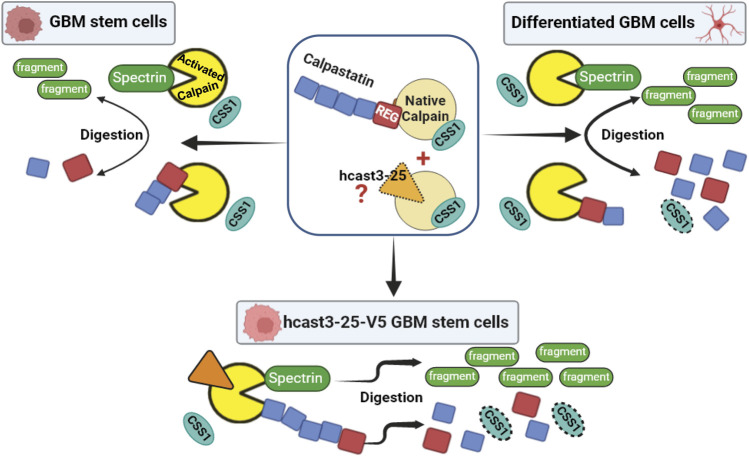
Proposed model for the mechanism of action of the calpain/calpastatin system in GBM stem cells, differentiated cells, and GBM stem cells overexpressing hcast-3-25 in light of current and previous results. In the center: Native inactive calpain (yellow) is associated to CSS1 (light blue) and to full length calpastatin (with four inhibitory units, blue) through the N-terminal regulatory domain (red). Hcast 3-25 protein (not detectable by WB, orange with dotted line) competes with full length calpastatin for binding to native calpain (De Tullio et al., 2018). In the stem condition when calpain is activated (bright yellow), CSS1 dissociates from the catalytic subunit. Calpastatin is cleaved by calpain with the liberation of free inhibitory units, thus allowing the inhibition of other activated calpain molecules (De Tullio et al., 2000; Stifanese et al., 2010). In this condition activated calpain can also cleave spectrin producing specific digestion fragments (Czogalla and Sikorski, 2005). Following serum differentiation, the increased activation of calpain induces enhanced calpastatin and CSS1 digestion whereas spectrin is only partially affected. The transfection of GBM stem cells with hcast 3-25-V5 results in an increased activity of calpains. Indeed hcast 3-25-V5 competes with endogenous calpastatins for binding to native calpain (Sparatore et al., 2019) but, being deprived of inhibitory units, leaves the active site of the protease free to digest calpastatin, CSS1 and spectrin.

In Figure 8 we propose a schematic model summarizing the calpain/calpastatin system in basal conditions considering GBM stem cultures, their differentiated counterparts, and hcast-3-25-overexpressing GBM stem cells. This model is based on previous experiments carried out *in vitro* (De Tullio et al., 2014; De Tullio et al., 2018; Sparatore et al., 2019) where we have observed that recombinant hcast 3-25 associates to native calpain competing for this binding with the Casts species containing the regulatory domain, and importantly, preserves the activity of calpain in the presence of Ca^2+^.

## Data Availability

The datasets presented in this study can be found in online repositories. The names of the repository/repositories and accession number(s) can be found below: GEO accession GSE179356.
