# Supplementary material for: Expression of calpastatin hcast 3-25 and activity of the calpain/calpastatin system in human glioblastoma stem cells: possible involvement of hcast 3-25 in cell differentiation
Source: Front Mol Biosci. 2024 Jul 30;11:1359956. doi: 10.3389/fmolb.2024.1359956 (PMC11319182; doi:10.3389/fmolb.2024.1359956)
Supplement: Supplementary file 1 [file DataSheet1.docx]

| Code# | Age | Sex | WHO grade | Status | Localization  (emisphere/lobe) | Tumorigenicity of GSCs (mice survival, days) |
| --- | --- | --- | --- | --- | --- | --- |
| GBM2 | 52 | M | IV | Primary | DX/P-O | yes |
| GBM3 | **48** | **M** | **IV** | **Primary** | **SX/T-P-O** | **120** |
| GBM4 | **78** | **M** | **IV** | **Secondary to WHO III astrocytoma** | **SX/P** | **yes** |
| GBM5 | **67** | **M** | **IV** | **Primary** | **DX/T-O** | **55** |
| GBM6 | 51 | M | IV | Primary | DX/T-P | 180 |
| GBM7 | 71 | M | IV | Primary | DX/F-T | 75 |
| GBM10 | 70 | F | IV | Primary | DX/F | 120 |
| GBM14 | 53 | M | IV | Primary | SX/P | ND |
| GBM15 | 57 | M | IV | Primary | - | yes |
| GBM19 | **40** | **F** | **IV** | **Secondary to oligodendroglioma** | **DX/F-T** | **100** |
| GBM23 | **70** | **F** | **IV** | **Primary, multicentric** | **SX/T-P-O** | **120** |
| GBM24 | **52** | **M** | **IV** | **Recurrent** | **SX/P** | **100** |
| GBM25 | 79 | M | IV | Primary | - | 240 |
| GBM30 | 68 | F | IV | Primary | - | ND |
| GBM37 | 73 | F | IV | Primary | - | 150 |
| GBM39 | **52** | **M** | **IV** | **Primary** | **-** | **100** |

**SUPPLEMENTARY MATERIAL**

**Expression of hcast 3-25 and activity of the calpain/calpastatin system in human glioblastoma stem cells: involvement of hcast 3-25 in differentiation**

**Table. S1 Clinico-pathological features of patients, glioblastomas (GBMs) and in vivo tumorigenicity of GBM-derived stem cells (GSCs)**

WHO= World health Organization, M=male, F= female, DX= right, SX= left, F= frontal, O= occipital, P= parietal, T= temporal ND= not determined. **GBMs used for experiments are reported in bold and grey rows.**

**
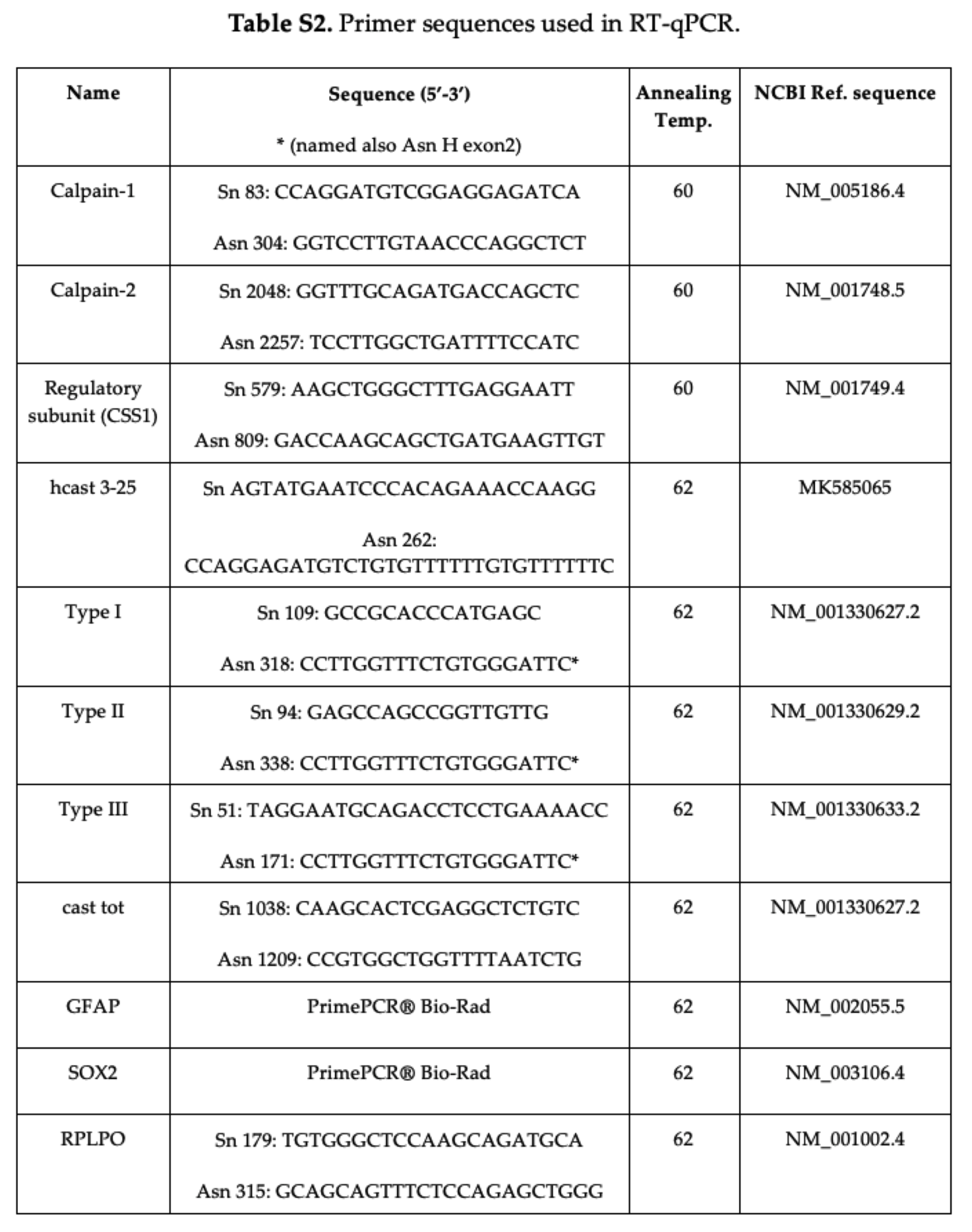
**

**
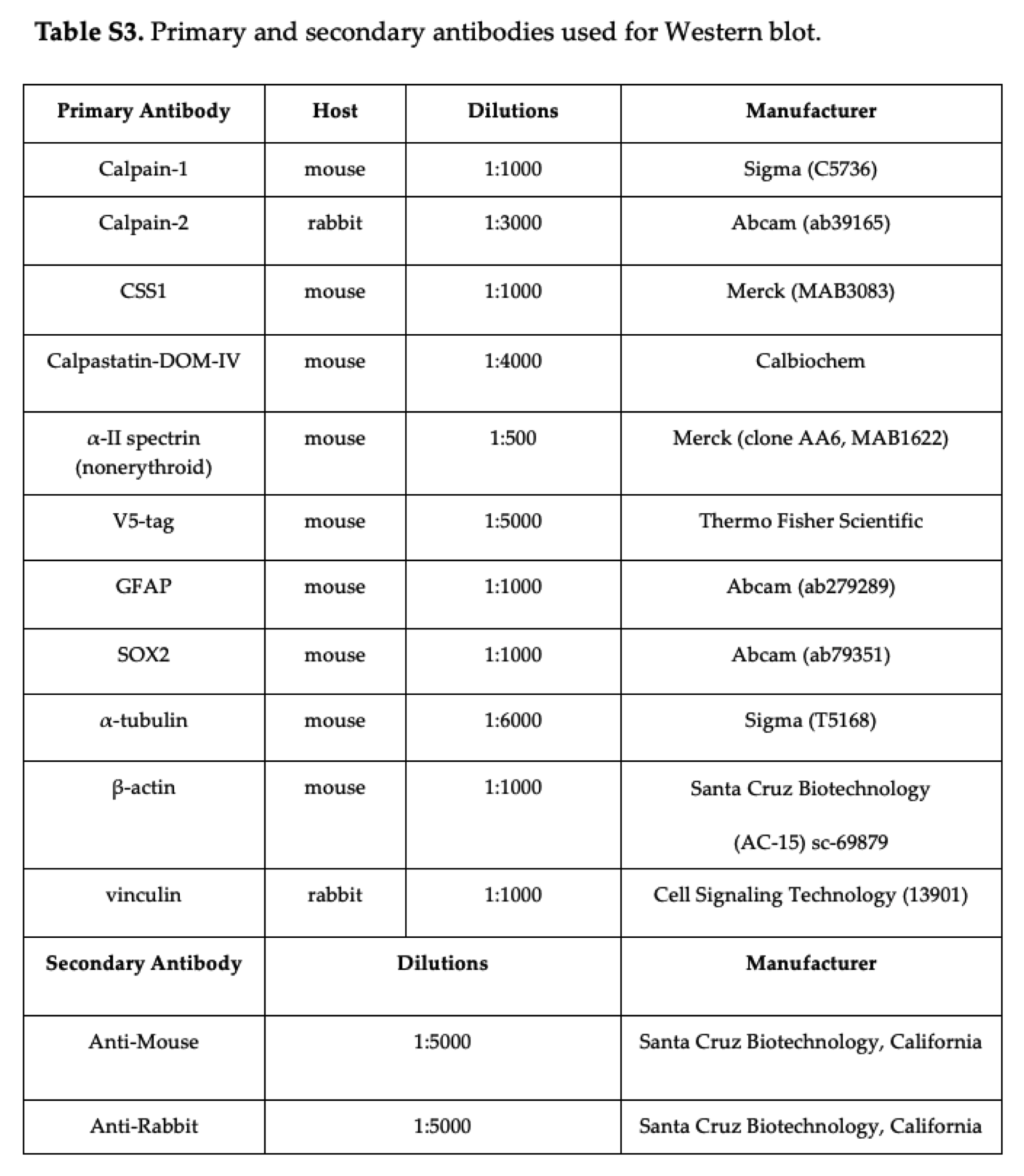
**

**Table. S4 Fold changes in mRNA expression of the calp/cast system components between stem and differentiated GBM cultures.**

|  | **GBM3 diff** | **GBM23 diff** | **GBM5 diff** | **GBM19 diff** |
| --- | --- | --- | --- | --- |
| **hcast 3-25** | 10,06 | 0,61 | 7,76 | 0,06 |
| **Type I** | 1,96 | 7,87 | 43,56 | 3,11 |
| **Type II** | 3,06 | 3,49 | 17,42 | 1,98 |
| **Type III** | 7,31 | 6,23 | 3,35 | 4,31 |
| **cast tot** | 1,90 | 2,47 | 5,53 | 2,72 |
| **Calp-1** | 33,66 | 4,79 | 60,57 | 4,69 |
| **Calp-2** | 2,27 | 23,61 | 31,62 | 1,81 |
| **CSS1** | 13,84 | 7,09 | 91,48 | 4,27 |

RT-qPCR was performed with specific primers targeted to the indicated genes. mRNA levels were measured as fold change in the expression of the indicated gene in GBM differentiated cells compared to their matching stem counterpart. Values are normalized against RPLPO, used as housekeeping gene. The fold change was calculated using the 2-^ΔΔCt^ method *vs.* respective stem culture.

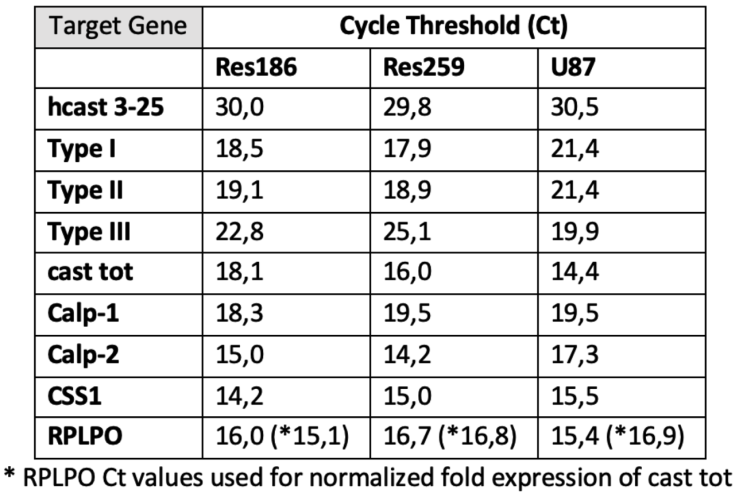


**Figure S1. mRNA levels of the calp/cast system components in Res186, Res259, and U87 human glioma cell lines.** Total RNAs were isolated from each sample and RT-qPCR was performed with specific primers targeted to the indicated genes. mRNA expression was measured as ΔCt versus RPLPO, used as housekeeping gene. Bars represent the mean ± SD from 5 experiments. Table reports Cycle threshold values (Ct) for the genes considered.

**
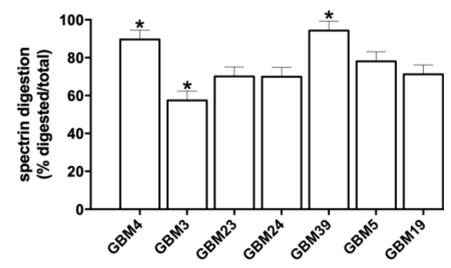
**

**
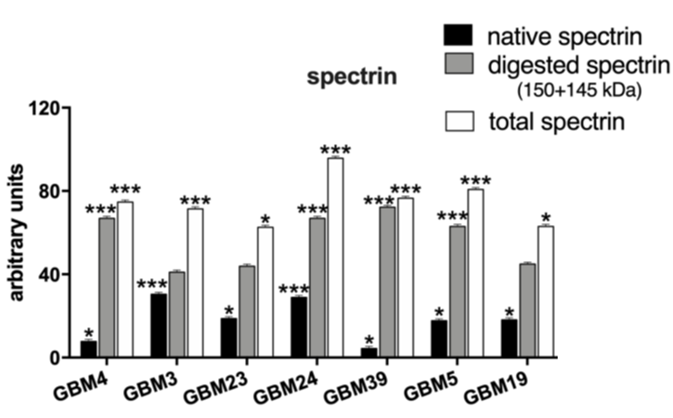
**

**Figure S2.** **Protein levels of α-II spectrin in GBM stem cells.** Proteins from crude lysates (10 μg) prepared from the indicated GBM stem cultures were separated by SDS/PAGE (10%) and submitted to immunoblotting. Native (240 kDa) and digested α-II spectrin (150+ 145 kDa) forms are indicated by black arrows and α-tubulin was used for protein loading control and normalization. Res 186 are reported as running condition control. *Histogram:* **upper**: α-II spectrin digestion is expressed as percentage of digested versus total protein. **lower:** quantification of the immune-reactive bands corresponding to native (black bars), digested (grey bars) and total α-II spectrin (white bars). Bars are the mean values of densitometry analyses ±SD from 3 replicates. Densitometry analysis for protein levels were normalized to α-tubulin and data are expressed as arbitrary units. *p<0.05 and ***p<0.001 by unpaired, two-tailed t-test.


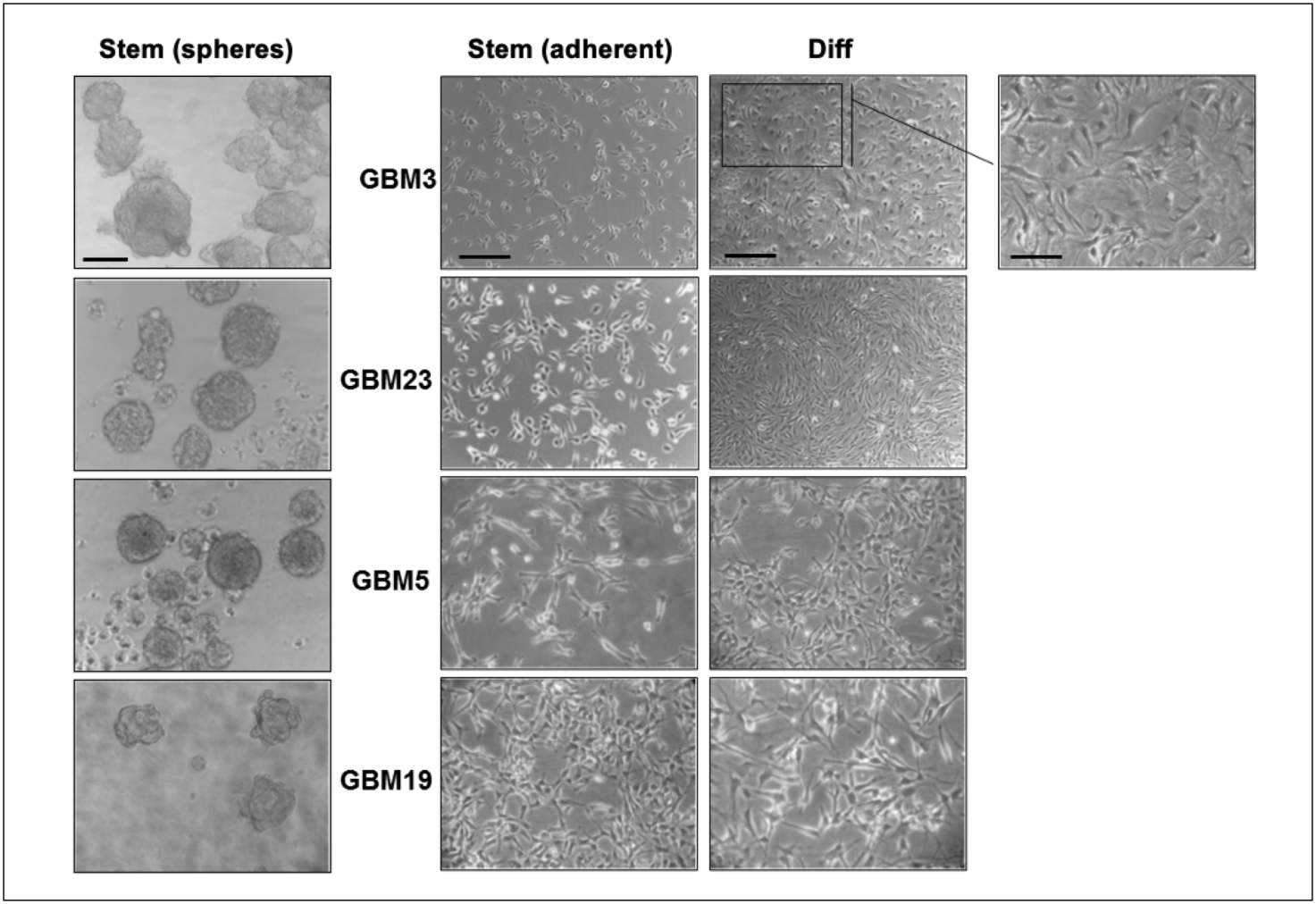


**Figure S3. Representative microphotographs of stem and differentiated GBM culture morphology**. GBM stem (Stem) cells grow as spheres (clusters enriched in cancer stem cells) in the absence of extracellular matrix (Matrigel) coating (scale bar: 150μm), while they adhere to the substrate as monolayer on Matrigel-coated substrates (scale bar: 50μm). GBM stem cells were shifted to FBS-containing medium for 15 days to obtain differentiated counterpart. In these conditions cells adhere to substrate without Matrigel (scale bar: 50μm). The inset details the presence of multipolar cells with signs of branching in differentiated cells (scale bar: 20μm).

**Figure S4. Protein levels of GFAP and SOX2 in stem and differentiated GBM3, GBM23, GBM5, GBM19 cultures.** Representative WB of GFAP, SOX2 and β-actin used as protein loading control. The histograms represent the densitometry quantification normalized to β-actin.


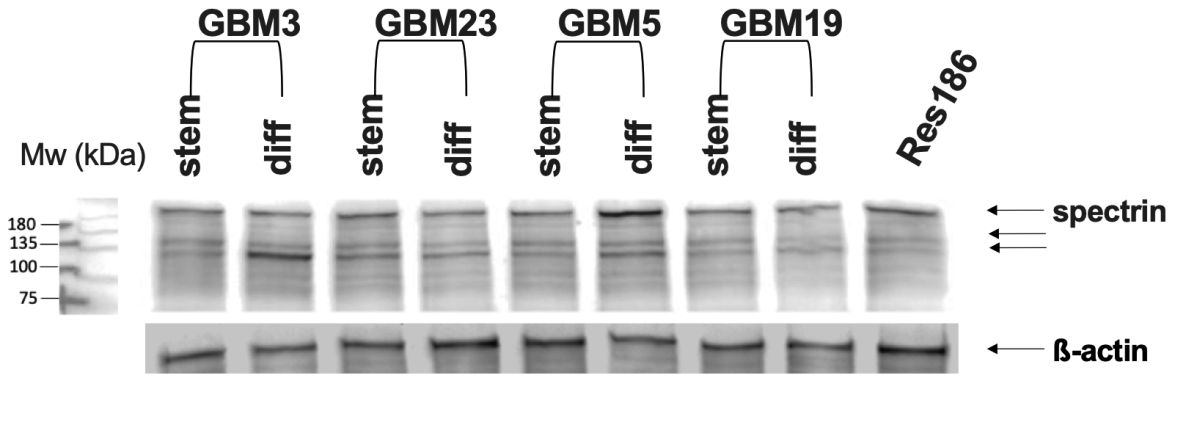

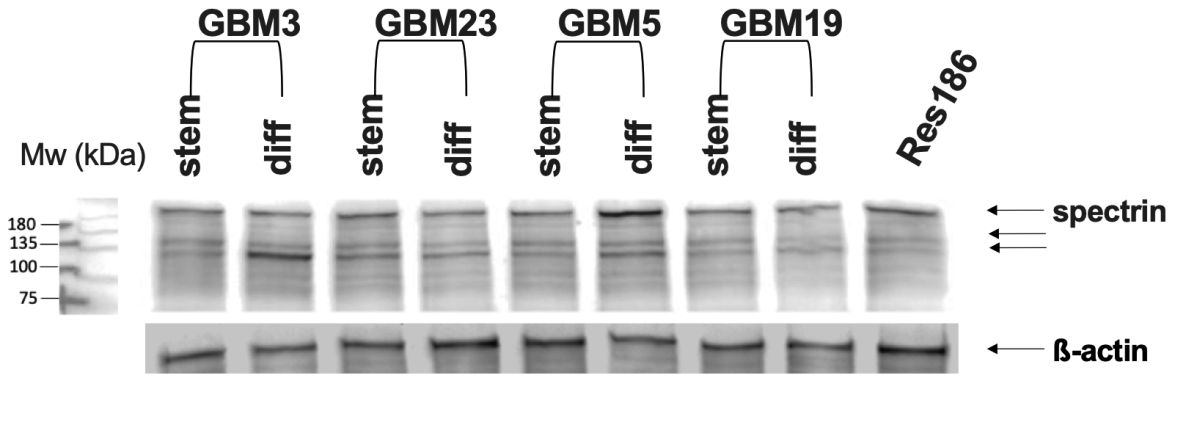


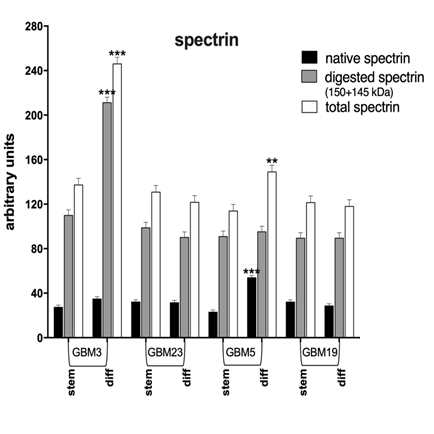


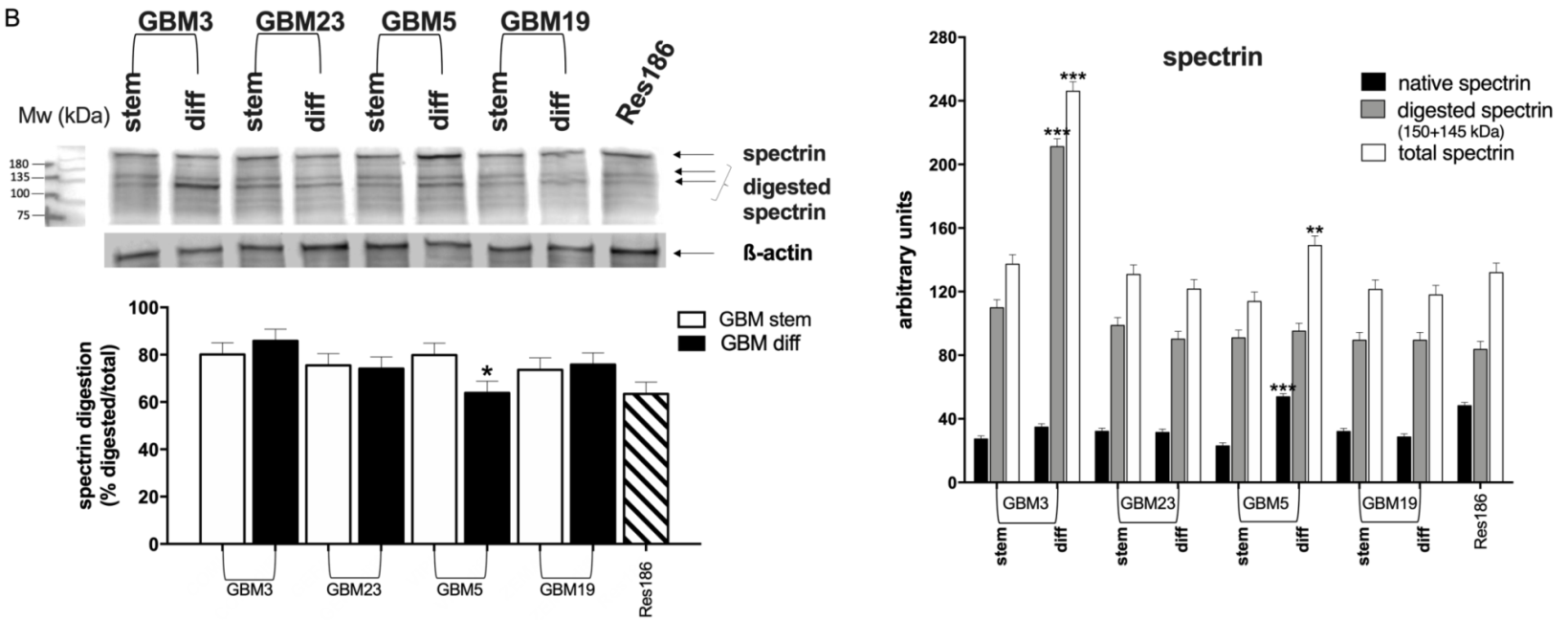

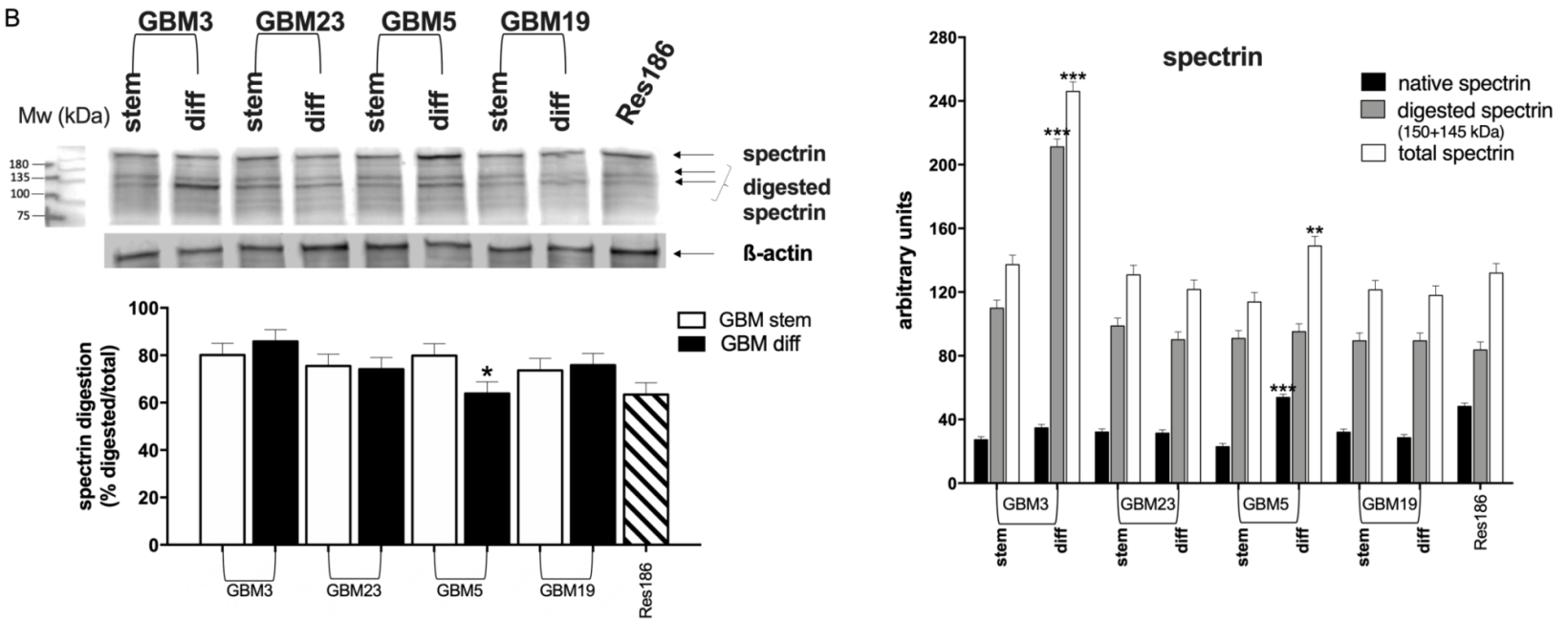


**Figure S5.** **Comparison of protein levels of α-II spectrin between stem and differentiated GBM cultures.** Proteins from crude lysates (10 μg) prepared from the indicated GBM stem and matched differentiated cultures were separated by SDS/PAGE (10%) and detected by immunoblotting. Native (240 kDa) and digested α-II spectrin (150+ 145 kDa) forms are indicated by black arrows and β-actin was used as protein loading control. *Left histogram:* α-II spectrin digestion expressed as percentage of digested versus total protein. *Right histogram*: protein quantification of the immune-reactive bands corresponding to native (black bars), digested (grey bars) and total α-II spectrin (white bars). Bars are the mean values of densitometry analyses ±SD from 3 replicates. Densitometry analysis for protein levels were normalized to β-actin (detected without stripping, in the same gel) and data are expressed as arbitrary units. **p<0.01 and ***p<0.001 *vs.* the respective GBM stem culture by unpaired, two-tailed t-test.
